# Experimental determination of three-dimensional cervical joint mobility in the avian neck

**DOI:** 10.1186/s12983-017-0223-z

**Published:** 2017-07-24

**Authors:** Robert E. Kambic, Andrew A. Biewener, Stephanie E. Pierce

**Affiliations:** 1 Museum of Comparative Zoology and Department of Organismic and Evolutionary Biology, Harvard University, Cambridge, MA 02138 USA; 2 Concord Field Station, Department of Organismic and Evolutionary Biology, Harvard University, Bedford, MA 01730 USA

**Keywords:** Function, Mobility, XROMM, Avian, Bird, Anatomy, Cervical, Neck, X-ray, Motion, Morphology

## Abstract

**Background:**

Birds have highly mobile necks, but neither the details of how they realize complex poses nor the evolution of this complex musculoskeletal system is well-understood. Most previous work on avian neck function has focused on dorsoventral flexion, with few studies quantifying lateroflexion or axial rotation. Such data are critical for understanding joint function, as musculoskeletal movements incorporate motion around multiple degrees of freedom simultaneously. Here we use biplanar X-rays on wild turkeys to quantify three-dimensional cervical joint range of motion in an avian neck to determine patterns of mobility along the cranial-caudal axis.

**Results:**

Range of motion can be generalized to a three-region model: cranial joints are ventroflexed with high axial and lateral mobility, caudal joints are dorsiflexed with little axial rotation but high lateroflexion, and middle joints show varying amounts axial rotation and a low degree of lateroflexion. Nonetheless, variation within and between regions is high. To attain complex poses, substantial axial rotation can occur at joints caudal to the atlas/axis complex and zygapophyseal joints can reduce their overlap almost to osteological disarticulation. Degrees of freedom interact at cervical joints; maximum lateroflexion occurs at different dorsoventral flexion angles at different joints, and axial rotation and lateroflexion are strongly coupled. Further, patterns of joint mobility are strongly predicted by cervical morphology.

**Conclusion:**

Birds attain complex neck poses through a combination of mobile intervertebral joints, coupled rotations, and highly flexible zygapophyseal joints. Cranial-caudal patterns of joint mobility are tightly linked to cervical morphology, such that function can be predicted by form. The technique employed here provides a repeatable protocol for studying neck function in a broad array of taxa that will be directly comparable. It also serves as a foundation for future work on the evolution of neck mobility along the line from non-avian theropod dinosaurs to birds.

**Electronic supplementary material:**

The online version of this article (doi:10.1186/s12983-017-0223-z) contains supplementary material, which is available to authorized users.

## Background

Birds are one of the most prominent groups of vertebrates in modern ecosystems. They are both taxonomically (~10,000 species) and ecologically diverse, inhabiting varied environments around the Globe. Their derived, feather-covered bodies have been a source of research inspiration for generations, with studies documenting macroevolutionary patterns (e.g. [1–6]), adaptation and selection (e.g. [7–9]), and functional morphology and development (e.g. [10–12]). The acquisition of the avian bauplan from non-avian theropod dinosaurs is detailed by a rich fossil record which illustrates an accumulation of ‘bird-specific’ characteristics – e.g. feathers, pneumatised skeleton, unidirectional breathing – through evolutionary time [13–15]. One distinctively avian feature is the highly mobile neck; an individual preening may move its head upside-down and back to reach its tail, place its head underneath its wing, and then move the head along its belly, all in a series of smoothly coordinated maneuvers. Despite their striking mobility and morphology, bird necks are relatively under-studied compared to the appendicular skeleton and skull [16, 17], although recent work has started to address this (e.g. [18–20]). As a result, neither the function nor the evolution of this complex musculoskeletal system is well-understood.

Anatomically, bird necks are interesting as they vary in cervical count from 9 to 25, although 14-15 is more typical [21–23]. Additionally, the morphology of the centra is unusual: they are heterocoelous (saddle-shaped) which is only seen elsewhere in pleurodire turtles [24, 25]. Functionally, avian necks are typically considered to have substantial mobility in dorsoventral flexion and lateroflexion, while the heterocoelous centra are accepted to preclude significant axial rotation (torsion) in vertebrae caudal to the atlas/axis [23, 26–28]. Quantification of avian cervical joint range of motion (ROM) has frequently been performed passively via hand-manipulation of cadaveric material with and without soft-tissues intact [17, 26, 27, 29–35]; however, a few studies have documented in vivo neck motion (e.g. [17, 32]). Most prior work has focused on a single degree of freedom: dorsoventral flexion [17, 29–32, 36–38]. A few studies have quantified lateroflexion in addition to, and independently of, dorsoventral flexion [26, 33]. Only three studies have attempted to reconstruct bird neck movements in three dimensions (specifically in owls [35, 39, 40]), with the potential to quantify all three rotational degrees of freedom simultaneously, including axial rotation. Such data are critical for understanding joint function, as musculoskeletal movements incorporate motion around multiple degrees of freedom simultaneously (e.g. [34, 41–44]).

Precise 3-D data on cervical joint motion would help illuminate how birds realize complex neck poses and how articular facets (zygapophyses) interact at different neck configurations. Historically, it has been difficult to measure the 3-D position of an intact joint with overlying soft tissues, but recent advances in X-ray-based techniques, specifically X-Ray Reconstruction of Moving Morphology (XROMM, [45, 46]), have opened the door to quantifying complicated joint motions. Here we use XROMM to reconstruct 3-D cervical joint function in the neck of a generalist bird – the wild turkey (*Meleagris gallopavo)* – with soft tissues intact.

We sought to address a number of questions: (1) Is the bird neck divisible into morphofunctional zones? Prior studies have commonly identified three cervical regions within the avian neck (e.g. [26, 29, 31, 33, 47]), consisting of a cranial (excluding the atlas and axis), middle, and caudal region; however, these regions do not have consistent divisions or mobility across taxa or studies [39]. (2) Do heterocoelous intervertebral joints limit axial rotation? Saddle joints are generally considered to be very mobile about two rotational axes, permitting flexion/extension, lateroflexion, and circumduction, while preventing axial rotation [48]. Previous research on avian necks has supported this interpretation [26–28], but axial rotation can be difficult to measure [42]. (3) Do zygapophyseal articulations maintain substantial overlap during complex motions? Percentage of zygapophyseal overlap (e.g. 50% overlap) has been used as a guide to reconstruct ROM in osteological specimens (including fossils) [49–53]; yet data is currently lacking on the degree of overlap in intact intervertebral joints [54]. (4) Are there complex interactions among degrees of freedom at intervertebral joints? Intuitively, one degree of freedom reaching maximum excursion often limits rotation about other axes (e.g. [26, 55, 56]). Measuring motion about multiple axes simultaneously may recover more complicated interactions [57].

## Methods

### Subjects and experimental setup

Five frozen wild turkey cadavers were acquired from Massachusetts Fisheries and Wildlife and data were collected on these specimens after thawing. The remains were accessioned into the Ornithology Department at the Museum of Comparative Zoology after data collection and CT scanning was complete (MCZ numbers 364027, 364028, 364029, 364460, 364461). We selected wild turkeys as the study organism for a number of reasons. First, they are easy to acquire and are commonly used in studies of functional anatomy (e.g. [58, 59]). Secondly, they are large birds with large cervical vertebrae that were amenable to implantation with radiopaque markers (see below). Thirdly, their cervical count (14 including atlas and axis) is average for birds. Finally, turkeys do not have highly specialized necks for unusual feeding behaviors.

In order to track intervertebral joint kinematics, radiopaque stainless steel beads 1.258 mm in diameter (Model # 55985, Bal-tec, Los Angeles, CA, U.S.A.) were implanted into the cervical vertebrae. Implantation was performed by dissecting down to bone, and then using a custom-machined carbide rod held in a pin vice to drill a well for the bead, which was then inserted and affixed with superglue. We attempted to minimize muscle damage and keep the soft tissues of the neck as intact as possible during this procedure. Ligaments and tendons were untouched, and muscles were separated along fascial planes using blunt dissection to access the bone. Despite these precautions, there was likely some localized damage to the muscles near implantation sites due to the increased stiffness of cadaveric tissue, but this should not have significantly affected our results. The atlas was not implanted due to its small size, its radically different morphology as compared to the other vertebrae, and the presence of capital muscle sheets that prevented non-destructive access to the bone. Although we attempted to implant the axis, we did not have three reliable markers stay in the bone in any of the specimens, which is required for marker-based XROMM. Thus, cervical vertebra three (C3) was the most cranial vertebra that could be reliably marked and tracked. Although turkeys have 14 cervical vertebrae, the pectoral girdle prevented access to C12-C14.

Once implanted with radiopaque beads, the cranial end of the neck was secured to a wooden stick with twine or zip-ties to allow manipulation while keeping hands free of the X-ray field. For two specimens, the caudal end of the neck was dissected away from the thorax to ensure that caudal joints achieved full excursion. In these cases, the caudal end of the neck was also secured to a piece of wood. The necks were then hand–manipulated (see further below) within an overlapping X-ray volume that was formed by the intersection of two C-arm X-ray fluoroscopes (Model 9400, OEC-Diasonics Inc.; modified by Radiological Imaging Services) aligned ~90 deg. to each other. ROM manipulations were simultaneously recorded using two Photron Fastcam 1024 PCI cameras (Photron USA Inc., San Diego, CA, USA) that each filmed the output of their respective fluoroscope. Videos were recorded at 1024 × 1024 resolution, at 60 frames per second with a shutter speed of 1/125 s (Fig. 3).

### ROM manipulation

Each neck was posed in a variety of configurations, some intended to maximize the excursion of a single degree of freedom, and others to capture intervertebral motions during complicated poses. The configurations included a mix of realistic poses which might be obtained in vivo, and poses which an animal would not naturally adopt but would reach ROM limits; starting poses were also a mix of realistic and unlikely. Simple motions included dorsiflexion, ventroflexion, and lateroflexion. More complicated poses included axially rotating the head and allowing the neck to follow, “looking over the shoulder”, and other combinations of lateroflexion, dorsoventral flexion, and axial rotation. The necks were typically so flexible that it was not possible to rotate a degree of freedom to its extreme along the entire length simultaneously. Therefore, for simple motions, such as dorsiflexion or lateroflexion, one trial maximizing the cranial joints and one maximizing the caudal joints were recorded. For these trials, a subset of the neck vertebrae would be secured, allowing the extra length to hang loose.

### Anatomical coordinate systems

Once data collection for a specimen was complete, the vertebrae were disarticulated and frozen for CT scanning. Joint capsules were examined during disarticulation to ensure that the ROM manipulations had not damaged them. Scan data were acquired on a Skyscan 1173 μ-CT (Bruker Corporation, Billerica, MA, USA) at 1120 × 1120 resolution and 71.05 μm voxel size. Scans were segmented and bone models were generated using Mimics v.17 (Materialise, Leuven, Belgium). Bone models were converted from .stl to .obj with MeshLab (Visual Computing Lab - ISTI – CNR, www.meshlab.net) and imported into Maya 2014 (Autodesk, San Rafael, CA, USA).

**Fig. 1:**
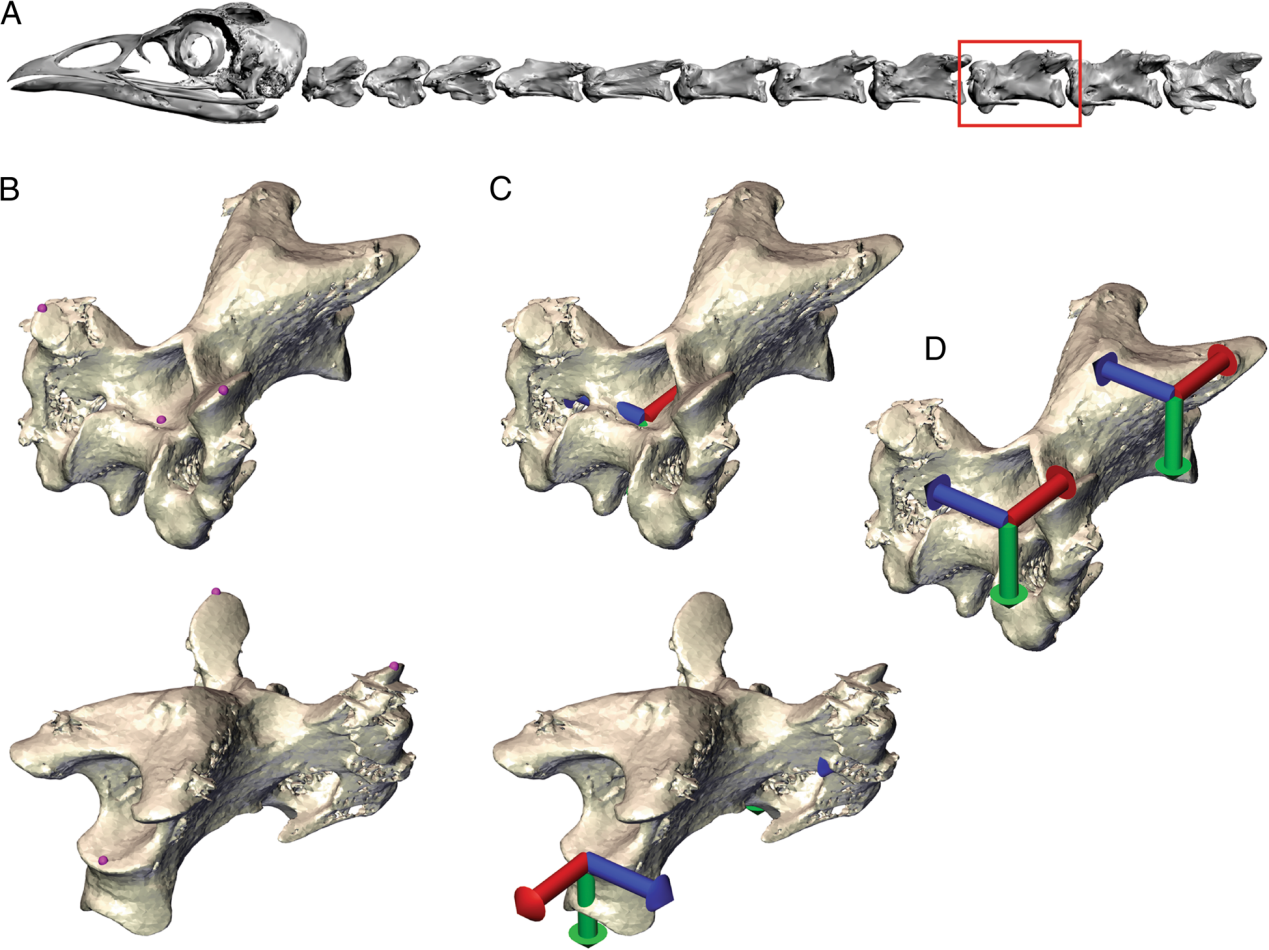
Building anatomical coordinate systems. **a**. Reconstructed left lateral view of the skull and neck with C10 boxed. **b-d**. Dorsolateral views of C10. **b**. Cranial (*top*) and caudal (*bottom*) views of C10 with *purple* spheres marking the points used to build the horizontal and axial vectors. **c**. Views as in **b**. of C10 with anatomical coordinate systems in place. **d**. Cranial view of C10 with anatomical coordinate systems made more visible

Anatomical coordinate systems were established for use in measuring joint motion (Fig. 1). Two vectors were used to create 3-D axes for the cranial and caudal joints of each vertebra: a horizontal vector was created by manually identifying the dorsal-most point on each prezygapophysis; and an axial vector was created by manually identifying a ventral midline point of the vertebral foramen at the cranial and caudal ends of the vertebrae, just proximal to the articular faces of the vertebral body. The 3-D coordinate axis was created by crossing the horizontal and axial vectors to calculate an orthogonal vertical axis, and then crossing the axial vector with the vertical vector. This last step produced an improved horizontal vector that was guaranteed to be orthogonal to the axial vector. These three vectors defined the anatomical coordinate system for each joint. The origins of the two anatomical coordinate systems (one for the cranial joint and one for the caudal) were placed at the points used to define the axial vector (Fig. 1c, d). The relative motion between the caudal anatomical coordinate system of the cranial vertebra and the cranial anatomical coordinate system of the caudal vertebra was calculated as a joint coordinate system [60].

**Fig. 2:**
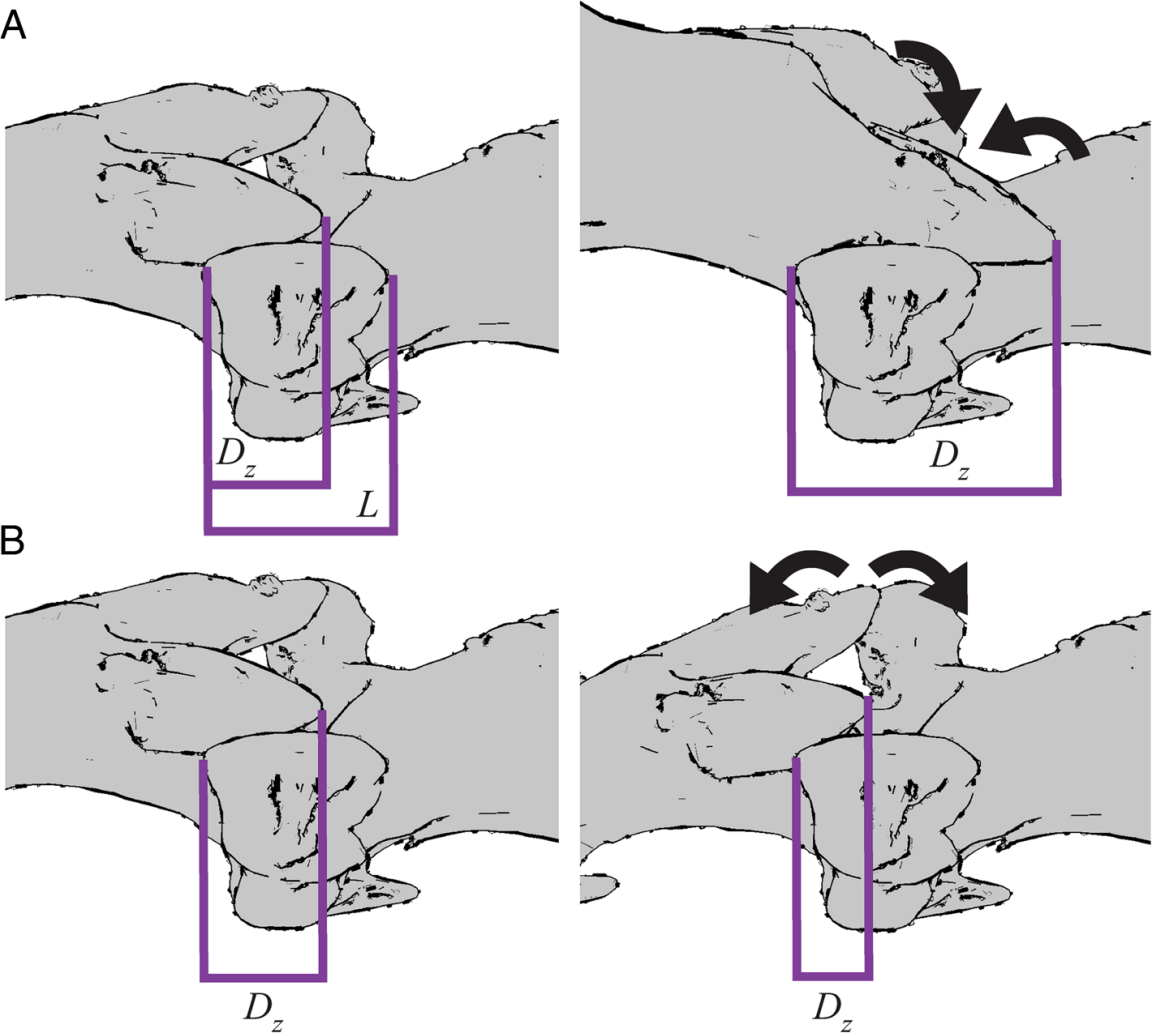
Measuring zygapophyseal overlap. Left dorsolateral view of two articulating vertebrae. **a**. Distance change during dorsiflexion, where the zygapophyses are pushed together. **b**. Distance change during ventroflexion, where the zygapophyses are pulled apart. Distance measurements represented in *purple*. *L*: The length of the prezygapophysis; used to standardize the distance measurement (*D*_*z*_). *D*_*z*_: The amount of postzygapophyseal overlap with reference to the prezygapophysis

To examine zygapophyseal movements and determine the degree of overlap during ROM, we created an interactive (‘real-time’) distance measure in Maya (Fig. 2). Locators were placed at the caudal end of the postzygapophysis and cranial end of the prezygapophysis, with their separation measured using the built-in distance tool. Distance was standardized by converting to a percentage using the formula $\frac{D_z}{L}\times 100\%$, where *D*_*z*_ was the output of the distance tool, and *L* was the total length of the prezygapophysis. By this measure, complete overlap occurred at 100%, half overlap occurred at 50 or 150%, and non-overlap occurred at 0 or 200%, depending on the direction of the motion. A custom Maya script was created to perform this percentage conversion for every frame that had motion data for the joint.

### Data analysis

**Fig. 3:**
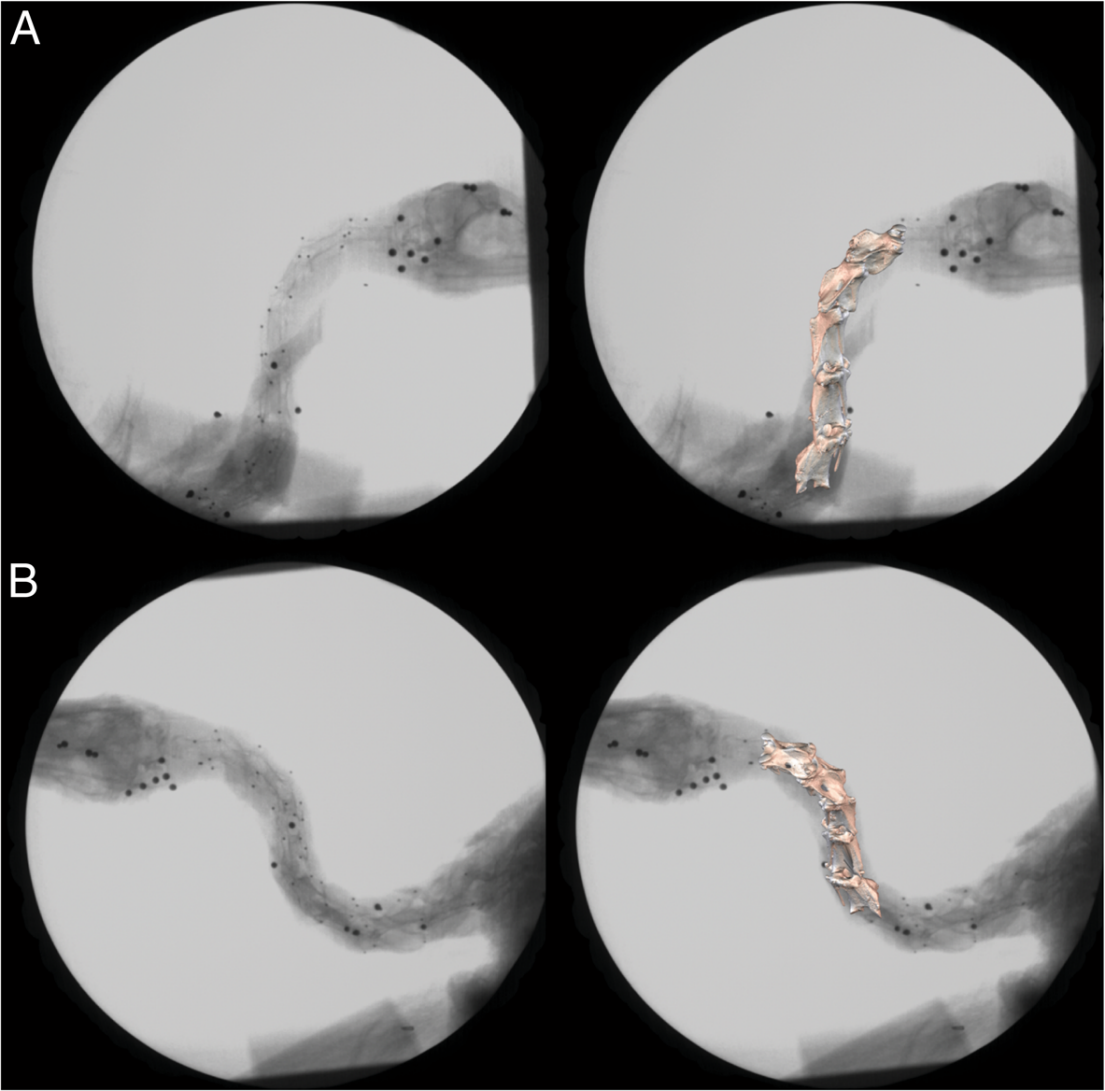
Stills from biplanar X-ray videos. **a**, **b**. *Left*: Original frame. *Right*: Frame with reconstructed bone motion overlaid

X-ray videos were analyzed using the marker-based XROMM workflow (www.xromm.org, [47]). XMA Lab 1.3.2 [61] was used for distortion correction, 3-D calibration, marker tracking, rigid body calculation, and filtering. A Butterworth filter with a frequency cutoff of 5 Hz was applied to rigid body animations to reduce noise. Rigid body kinematics was used to animate bone models in Maya using the XROMM Maya tools scripts (Fig. 3, www.xromm.org). Custom scripts were used to calculate relative motion between vertebrae as a joint coordinate system with a rotation order of XYZ. Dorsiflexion/ventroflexion served as the Z-axis, axial rotation as the X-axis, and lateroflexion as the Y-axis.

### Form vs. function

**Fig. 4:**
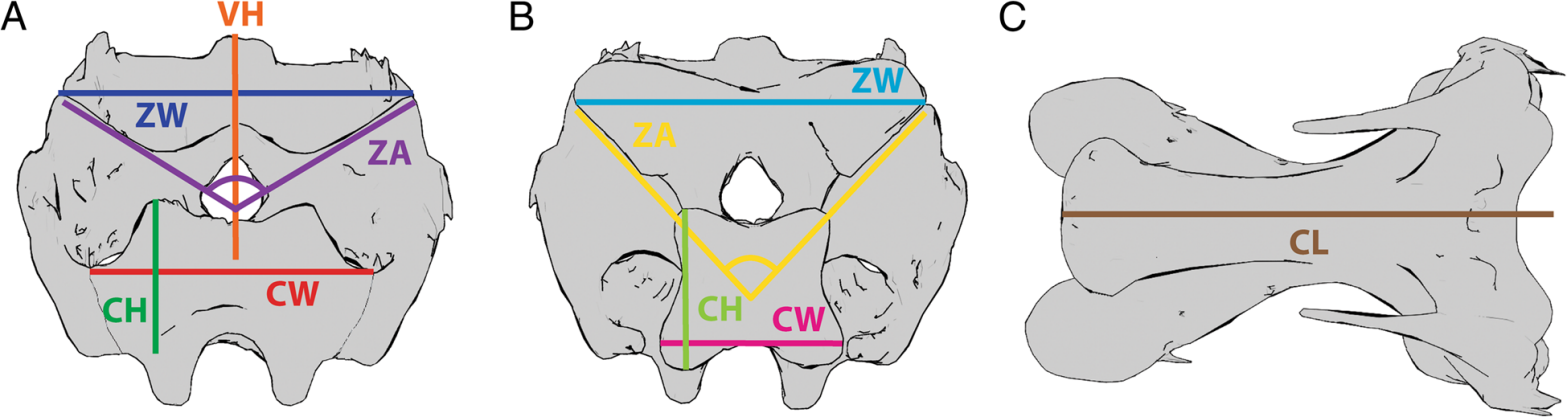
Measurements for morphometrics. Cervical vertebra in cranial (**a**), caudal (**b**), and ventral (**c**) views with linear measurements. CH: Centrum height. CL: Centrum length. CW: Centrum width. ZA: Zygapophyseal angle. VH: Vertebral height. ZW: Zygapophoseal width

To investigate the relationship between cervical morphology and function, we took linear and angular morphological measurements [Additional file 1] on vertebrae C3 through C12 for all five individuals (Fig. 4). These included length of the vertebral body (excluding zygapophyses) and height from the middle of the centrum to the most dorsal spine/surface. Measurements repeated for the cranial and caudal ends of the vertebra included the height and width of the articular facets of the centra, width of the zygapophyses measured from their most lateral points, and angle between the articular surfaces of the zygapophyses. Distance measurements were standardized by the cube root of the volume of C3 to account for body size differences (body weight could not be used as some specimens were incomplete when received). We then performed a principal component analysis on the standardized measurements and angles using the correlation matrix. Finally, we used an Akaike information criterion stepwise method to regress the ROM results on the first three principal components (PCs) [62]. The remaining PCs explained <5% of the variance in the data, and were not analyzed further. Two sets of regressions were performed: the first was on the maximum excursion of each degree of freedom, and the second was on the mean angular value of dorsoventral flexion. The latter was calculated as the average of maximum dorsiflexion and maximum ventroflexion; since they differ in sign, a joint which could ventroflex and dorsiflex the same amount would have a mean of zero. Regressions on mean axial rotation and lateroflexion angles were not performed as their ROM was expected to be symmetrical. Since an intervertebral joint is composed of two vertebrae, regressions were run separately with a vertebra modeled as contributing to the cranial joint or the caudal joint. Statistical analyses were run in R using the stepAIC function in the MASS package (R Core Team, www.R-project.org, [63]).

## Results

### ROM

**Fig. 5:**
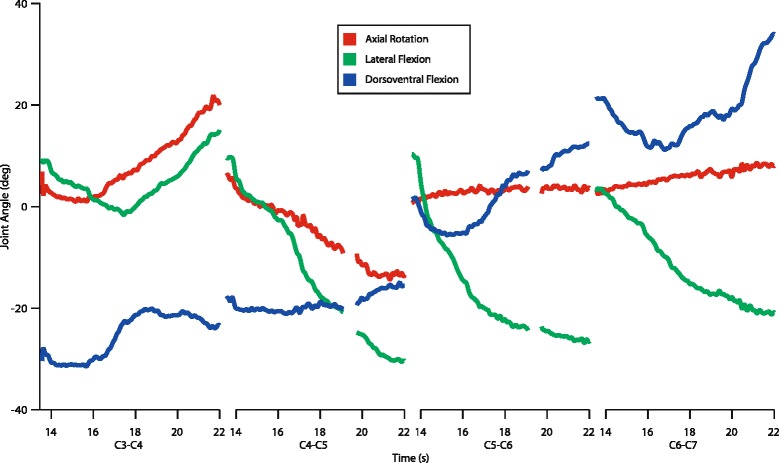
Joint angles for four cervical joints through time. In this trial, the neck was axially rotated from the cranial end over eight seconds. Joints are labeled according to the two cervical vertebrae they lie between, so C3-C4 corresponds to the motion measured between C3 and C4. Trace colors are *red*: axial rotation, *green*: lateral flexion, *blue*: dorsoventral flexion


Twenty-nine sequences from five individuals were analyzed, totaling 7667 frames of joint poses. Figure 5 shows a representative trial of the motions measured by the joint coordinate systems. In this trial, the caudal end of the neck was stabilized while the head was axially rotated, creating a helical winding of the neck as a whole [Additional file 2]. Such motion was designed to examine maximum range of movement at intervertebral joints, rather than mimicking in vivo neck motions (which have yet to be fully quantified). As is illustrated, every intervertebral joint dorsiflexed (Fig. 5; blue line), although the posterior joints (C5-C6: 14 deg., C6-C7: 13 deg) dorsiflexed more than the more cranial joints (C3-C4: 4 deg., C4-C5: 5 deg). All joints lateroflexed substantially (Fig. 5; green line), with the most cranial joint (C3-C4) lateroflexing less than the others (7 deg. versus 41, 35, and 23 deg). However, not every joint axially rotated (Fig. 5; red line). The two posterior joints (C5-C6 and C6-C7) underwent little to no axial rotation (2-3 deg), while the two cranial joints (C3-C4 and C4-C5) underwent substantial axial rotation (17 deg). This rotation appears to be tightly coupled with lateroflexion; note that the axial rotation and lateroflexion traces follow each other closely (Fig. 5).

Additional file 2: Render of the trial plotted in Fig. 5 in one X-ray camera view with animated bone models. Movie slowed by 2.4X. (MP4 3318 kb)


**Fig. 6:**
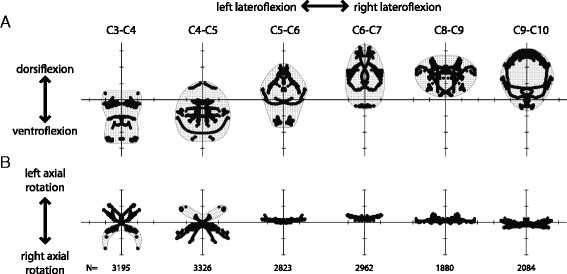
Plots of two degree of freedom ROM. Poses reached by the joint are plotted with *black circles*. **a**. Lateroflexion versus dorsoventral flexion. **b**. Lateroflexion versus axial rotation. Stippling indicates unmeasured poses that are hypothesized to be reachable. Ticks are set 20 deg. apart. Sample size in frames is indicated along the bottom

Combining every pose from all analyzed trials produced ROM volumes that reflect the potential rotations available to each joint (Fig. 6). Total range of dorsoventral flexion is similar across all joints, ~70 deg. (Fig. 6), although C8-C9 is the exception, allowing a smaller amount of dorsoventral flexion than the surrounding joints. While the range of dorsoventral mobility is similar across joints, the absolute values of dorsoventral flexion are regionalized. Cranial joints are more ventroflexed and caudal joints are more dorsiflexed. All joints allow lateroflexion (Fig. 6a, b), but cranial and caudal joints allow more lateroflexion (mean = 69 deg) than do C5-C6 or C6-C7 (54 deg., 43 deg). The most notable difference is in axial rotation (Fig. 6b). C3-C4 and C4-C5 permit substantial axial rotation (54 deg., 44 deg), in contrast to the limited axial rotation measured in more caudal joints (<12 deg).

### Zygapophyseal overlap

**Fig. 7:**
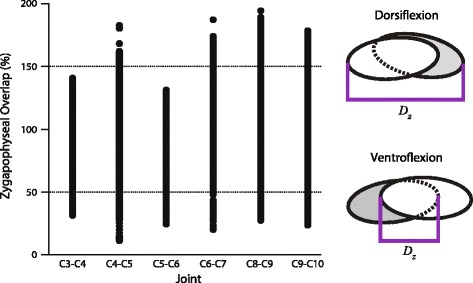
Zygapophyseal overlap for six joints. Zygapophyseal overlap, measured in percentage, is plotted for *right* and *left* zygapophyses combined. *Right*: Schematics showing the method of measuring zygapophyseal overlap. *D*_*z*_:the distance measured between the two zygapophyses. For more detail, see Methods

Our measurements found that zygapophyses stay in contact with each other within a 50% overlap zone over a broad range of movements (Fig. 7). However, zygapophyses are not restricted to maintaining this overlap. Every joint’s motion includes poses that reduce zygapophyseal overlap to under 50%. All cervical joints examined can ventroflex to zygapophyseal overlap <50%, and some come close to disarticulation. Most of the joints examined can also dorsiflex to <50% overlap, except for C3-C4 and C5-C6 which appear more restricted.

### Cervical morphology

**Fig. 8:**
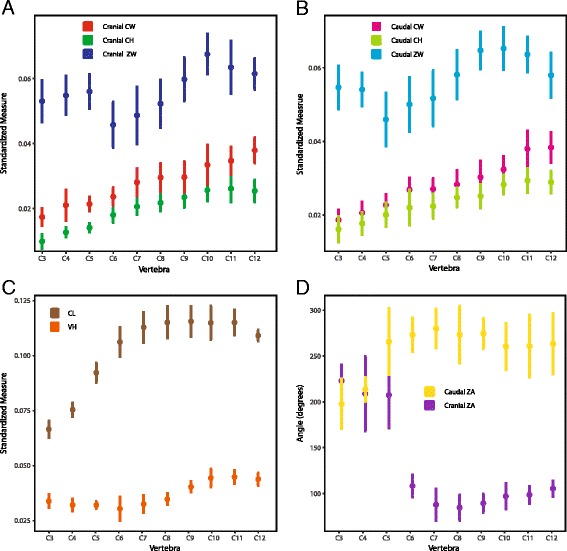
Standardized linear and angular measurements of turkey cervical vertebrae. Mean and standard deviations are plotted. Colors and abbreviations follow Fig. 4

Some standardized linear measurements of the cervical vertebrae plot as smooth cranial-to-caudal gradients, while others have rapid shifts at specific intervertebral joints (Fig. 8). For instance, centrum width and height increase gradually along the neck (Fig. 8a, b). In contrast, cranial and caudal zygapophyseal angles shift abruptly at C5 from flat, wide angles, to much narrower angles (Fig. 8d). Both pre and postzygapophyseal widths decrease at C5 and then increase to reach an inflection point at C10 (Fig. 8a, b).

**Fig. 9:**
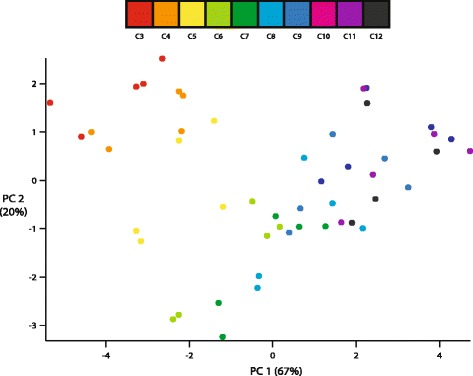
Scores for the first two principal components calculated for the cervical morphological measurements. The number in parentheses indicates the proportion of variance explained by each axis

A principal component analysis separates cervical vertebrae according to their position in the neck (Fig. 9). Plotting the first two principal components (PCs) generates a “U”-shaped plot; PC1 creates a cranial to caudal gradient, while PC2 separates out a middle region from cranial and caudal regions of the neck. PC1 loads centrum height and width, as well as vertebral height and length (Table 1). The linear measures are all loaded in the same direction, indicating that PC1 mostly captures size variation along the neck, with more caudal vertebrae enlarged compared to cranial vertebrae. PC2 positively loads zygapophyseal width, vertebral height, and prezygapophyseal angle. It negatively loads centrum length and postzygapophyseal angle. The loadings indicate that the middle section is composed of cervical vertebrae that have more narrowly set zygapophyses for their size, are elongate cranial to caudal, but short dorsal to ventral. PC3 positively loads postzygapophyseal width, and negatively loads pre- and postzygapophyseal angle and prezygapophyseal width. The variation PC3 captures does not separate cervical vertebrae along a cranial to caudal gradient, instead grouping such combinations as C4 and C5 with C11 and C12, and C3 with C7 and C8.

**Table 1:** Component loadings (eigenvectors) for the principal component analysis of vertebral measurements

Measurement	Component 1 (65.8%)	Component 2 (19.8%)	Component 3 (5.2%)
Caudal centrum height	**0.367937**	0.055284	−0.00162
Caudal centrum width	**0.364191**	−0.02796	−0.24035
Postzygapophyseal angle	0.18176	**−0.53805**	**−0.48951**
Postzygapophyseal width	0.286414	**0.384656**	**0.311566**
Cranial centrum height	**0.373541**	−0.08982	0.023154
Cranial centrum width	**0.362724**	−0.00474	−0.27056
Prezygapophyseal angle	−0.27875	**0.371415**	**−0.6021**
Prezygapophyseal width	0.257273	**0.461061**	**−0.34749**
Vertebral Height	**0.30735**	**0.322717**	0.143931
Centrum length	**0.327816**	**−0.30974**	0.166802

Percentage values indicate the proportion of variance explained by the axis. Variables loaded higher than .3 are bolded

Our regression analyses (Table 2) recovered a significant relationship between the first three PCs and both axial rotation excursion (r^2^ = 0.91, 0.77) and mean dorsoventral angle (r^2^ = 0.86, 0.90). PC1 and PC2 predicted lateroflexion excursion more weakly (r^2^ = 0.30, 0.50). Dorsoventral excursion was not significantly related to any PC axis (r^2^ = 0.09, 0.07).

**Table 2:** Regression results

Joint	Rotation	Variable	Coefficient	St. Err.	T value	*p*-value	Adj. r^2^	*p*-value
Cranial	axial rotation excursion	PC1 PC2 PC3	−5.8268 7.971 −6.5223	0.4825 0.7522 1.4763	−12.076 10.597 −4.418	<.001 <.001 <.001	0.9093	<.001
	lateroflexion excursion	PC1 PC2	−1.4410 6.112	0.6853 1.1422	−2.103 5.351	0.0449 <.001	0.5041	<.001
	dorsoventral excursion	PC2	−1.6532	0.8461	−1.954	0.0608	0.0886	0.06078
	mean dorsoventral flexion	PC1 PC2 PC3	6.6402 −5.8441 6.4364	0.6137 0.9567 1.8777	10.820 −6.108 3.428	<.001 <.001 0.0020	0.8627	<.001
Caudal	axial rotation excursion	PC1 PC2 PC3	−4.1545 7.6497 4.3237	0.8023 1.1911 2.2359	−5.178 6.422 1.934	<.001 <.001 0.0641	0.7681	<.001
	lateroflexion excursion	PC2 PC3	4.30274 3.955	1.3482 2.422	3.192 1.633	0.0037 0.1141	0.2957	0.003358
	dorsoventral excursion	PC3	−2.854	1.585	−1.801	0.0825	0.0718	0.08246
	mean dorsoventral flexion	PC1 PC2 PC3	4.9342 −7.6029 2.2219	0.5378 0.7984 1.4988	9.174 −9.522 1.482	<.001 <.001 .15	0.9025	<.001

Results of regressing range of motion on principal component scores for vertebrae. Each regression was run twice for the joint cranial to and then caudal to the vertebra. Rotation excursions are the differences between maximum and minimum angular values, while mean dorsoventral flexion is the average/midpoint between the maximum and minimum value. Only significant terms are shown. An adjusted R-squared and *p*-value is given for each model, and for each term the value of the coefficient, the standard error, and the t value and its *p*-value are given.

## Discussion

### Regionalization

**Fig. 10:**
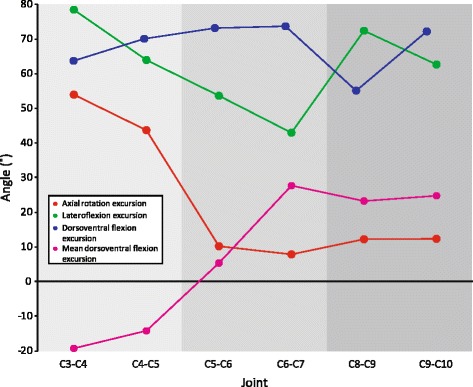
Axial rotation (*red*), lateroflexion (*green*), and dorsoventral flexion (*blue*) excursions and mean dorsoventral flexion (*pink*) excursion for the six sampled joints. Three potential regions highlighted with *shades of grey*

Prior studies on avian neck morphology/mobility have suggested that the cervical vertebrae are regionalized caudal to the atlas/axis complex, with a cranial, middle, and caudal region [26, 29, 31, 33, 40, 47]. These regions are not immediately apparent in the turkey neck; shifts in mobility occur at different joints depending on the rotation being considered. Dorsoventral flexion excursion is relatively constant, except for a dip at C8-C9 (Figs. 6 and 10). Mean dorsoventral excursion shifts from ventroflexed at C4-C5 to dorsiflexed at C6-C7, while C5-C6 is transitional and close to zero (Figs. 6 and 10). Lateroflexion ROM decreases steadily from C3-C4 to C6-C7, then increases at C8-C9 (Figs. 6 and 10). Axial rotation drops from substantial amounts at C3-C4 and C4-C5 to low amounts with no transitional joint (Figs. 6 and 10). The joints at which shifts in mobility occur vary, as does the presence of transitional joints that have intermediate amounts of mobility; this provides support for the argument that a fixed three region model for avian neck function is an oversimplification [39, 64].

Despite this variation, there are clusters of vertebrae with similar function. Cranial joints C3-C4 and C4-C5 axially rotate and are relatively ventroflexed. Caudal joints C8-C9 and C9-C10 are relatively dorsiflexed, allow little axial rotation, and are as mobile as C3-C4 and C4-C5 in lateroflexion. Between these lie middle joints C5-C6 and C6-C7 which vary in axial rotation, mean dorsoventral flexion angle, and have relatively lower lateroflexion mobility. Therefore, our data do appear to reflect three vertebral regions that have within-region variability in mobility. A PCA on maximum and minimum angles by joint supports this observation [Additional file 3]; vertebrae cluster into three regions when plotted by the first two PCs, but the distance between joints is close to the distance between regions.

### Dorsoventral and lateral mobility trends

In the turkey neck, cranial joints allowed the most ventroflexion, while caudal joints permitted the most dorsiflexion (Figs. 6 and 10); this pattern likely reflects the natural S-shaped curve of the neck. In contrast, dorsoventral flexion excursion (or absolute range of motion in the dorsoventral plane) remained relatively constant across the joints sampled (Figs. 6 and 10). This consistency likely leads to the failure of the regressions on dorsoventral excursion (Table 2), as morphology varied while excursion did not. A stable dorsoventral excursion most closely resembles results reported for chickens and ducks [29–31, 38], contrasting with results from ostriches where dorsoventral excursion increased in the middle portion of the neck [26, 33].

Lateroflexion mobility in turkeys appears most consistent with descriptions of mallard in Van der Leeuw et al. [31] and barn owls in Krings et al. [40]. Van der Leeuw et al. describe the maximum mobility of the mallard neck as being in the “most rostral part of the neck and in the caudal part of region 2”. Similarly, we observed the greatest amount of lateral mobility at C3-C4, decreasing lateral mobility moving caudally, and an increase at C8-C9 (Figs. 6 and 10). Barn owls were also inferred to have high mobility in cranial and caudal regions of the neck, with a decrease in the middle of the neck; unlike turkeys, owls had lower lateroflexion mobility overall including under 15 deg. of lateroflexion potential in the middle portion of the neck [40]. This pattern of lateroflexion ROM contrasts with those reported in cadaveric ostrich material with intact soft tissues. Dzemski and Chistian [26] recovered two zones of consistent excursions, roughly dividing the neck in two; the caudal half had greater flexibility than the cranial half. In contrast, Cobley et al. [33] describe a cranial region of low flexibility, a middle region of increased flexibility, and a caudal region of gradually decreasing flexibility. Neither pattern matches our results in turkeys, although it is unclear whether interspecific or methodological differences account for the discrepancy.

Another notable pattern in our data is that maximum lateroflexion does not occur at the same dorsoventral flexion angle across joints (Fig. 6). Lateroflexion is often measured from zero deg. dorsiflexion [26, 33], but in turkeys only some joints reach maximum lateroflexion at or close to zero deg. dorsiflexion (C3-C4, C5-C6); some need to be more dorsiflexed (C6-C7, C8-C9, C9-C10), and one needs to be more ventroflexed (C4-C5). The morphology that drives this interaction between dorsoventral flexion and lateroflexion may be similar in other taxa; Cobley et al. [33] noted that caudal joints in ostrich necks tended to dorsiflex when lateroflexed.

### Axial rotation

Surprisingly, our results do not support the hypothesis that axial rotation is restricted caudal to the atlas/axis. Even though the saddle-shaped centra are generally thought to prevent significant torsion [26–28], we measured 54 deg. of axial rotation in C3-C4 and 44 deg. in C4-C5 (Figs. 6 and 10). These joints compose the cranial region of the neck, marked by high axial rotation mobility, high lateroflexion mobility, and generally operating ventroflexed. Cranial joints with high axial and lateroflexion potential have also been reported in owls [40], suggesting this pattern of ROM is not tied to a specific avian clade. Axial rotation is coupled to lateroflexion; C3-C4 and C4-C5 can neither axially rotate without lateroflexing nor lateroflex without axially rotating (Figs. 5 and 6b). The two degrees of freedom interact such that it is not possible to measure axial rotation independent of lateroflexion. This likely explains prior results like those of Dzemski and Christian [26], who measured cervical ROM about a single axis at a time and reported almost no axial rotation outside of the atlas-axis joint. Similar “single axis” methods for estimating ROM are common when examining osteological specimens, especially fossils; our data suggest that such studies may have underestimated the degree of axial rotation possible at intervertebral joints.

We propose that prezygapophyseal morphology provides an explanation for the coupled lateroflexion and axial rotation. The prezygapophyses of C3, C4, and C5 are pitched forward compared to those of more caudal vertebrae (Additional file 3). Consider two vertebrae where the cranial vertebra is lateroflexing to the right. The postzygapophysis of the cranial vertebra on the right side will translate both caudally and dorsally as it slides along the inclined prezygapophysis with which it articulates. Meanwhile, the left postzygapophysis will either remain in its starting position or slide cranio-ventrally, depending on the location of the center of rotation. Such movement will impart both yaw towards the right as well as roll of the vertebral body, i.e. an axial rotation. Data from horses and humans [65–67] support the contention that pitched zygapophyses generate coupled rotations. Forward pitching of the prezygapophyses of cranial vertebrae may be common in birds, having been observed in an eagle, curassow, and pelican (REK pers. obs.), and described in the ostrich and owl [27]. Therefore, we predict that these taxa have the same coupled lateroflexion and axial rotation as turkeys.

### Zygapophyseal overlap

Maintenance of 25-50% overlap has been proposed as a guide for reconstructing cervical ROM in extinct taxa [49–51], based on the argument that the joint capsules restrict further motion. Other researchers have argued that this rubric may generally underestimate ROM [33, 54, 68], or overestimate lateroflexion [33]. Our results provide evidence that this method may underestimate cervical joint ROM in cadaveric and osteological specimens. Every zygapophyseal joint measured can move past 25% overlap in at least one direction (Fig. 7). When pulled apart, zygapophyses at C4-C5 reached only 11% overlap; when pushed together, zygapophyses at C8-C9 moved almost to disarticulation at 194% (=6%) overlap. Although living animals may maintain greater zygaphophyseal overlap to protect joint capsules (currently under investigation by the authors), we demonstrate that neither passive soft tissue constraints nor osteological stops restrict zygapophyses to 25-50% overlap during experimental cadaveric manipulation. Therefore, 25-50% zygapophyseal overlap would seem to represent a conservative estimate for cervical ROM.

### Form and function

The cervical morphology of turkeys varies along a cranial-caudal axis (Figs. 8 and 9) and is closely correlated to function. For instance, vertebrae C3, C4, and C5 are morphologically distinct (e.g. short centra with wide, low-angled prezygapophyses), and joints C3-C4 and C4-C5 are functionally distinct in allowing much more axial rotation than other vertebrae, and being the only ventroflexed joints when considering mean dorsoventral flexion (Fig. 10). Our form-function regressions further support this; both axial rotation and mean dorsoventral flexion are strongly predicted by the first three PC axes (Table 2), indicating that whole vertebral shape is important in determining patterns of mobility. However, this result makes it difficult to tease out which vertebral measures are most important in determining ROM. To investigate this further, we ran additional regressions on individual volume-standardized measurements [Additional file 3]; results demonstrate that a large suite of vertebral features, including centrum length, width, and height, and zygapophyseal angle and width, combine to predict axial rotation and mean dorsoventral flexion angle, again showing that overall vertebral shape impacts joint ROM.

Lateroflexion excursion was predicted by PC1 and PC2 (cranial joint) or PC2 and PC3 (caudal joint), but more weakly. Coefficient of determination values are lower than for axial rotation and mean dorsoventral flexion. PC2 is the principal component that appears in both regressions, and it is weighted much more heavily than PC1 in the cranial joint regression (Table 2). Four measures of zygapophyses are highly weighted in PC2 (Table 1), suggesting that zygapophyseal morphology factors heavily in lateroflexion mobility. Regressions based on individual morphological measurements [Additional file 3] support this contention; centrum and zygapophyseal width are the only measures that appear in both regressions.

Our results provide evidence that the unusual morphology of bird cervical vertebrae leads to unique form-function relationships. Other researchers have found that long centra allow more dorsiflexion and sometimes lateroflexion [69–73]. Turkeys do not appear to follow either of these patterns: centrum length increases from C3 to C7, then plateaus (Fig. 8c), but neither dorsoventral flexion nor lateroflexion results match this pattern. In mammals, increased height of centra has been suggested to restrict dorsoventral flexion [70, 73, 74]. The pattern is different in turkeys; centra gradually increase in height caudally (Fig. 8), but maximum dorsoventral flexion excursion remains relatively consistent (Figs. 6 and 10). Centrum width has been reported to restrict lateroflexion [18, 73, 74] while zygapophyseal width has been reported to restrict torsion [69]. Again, neither morphological trend matches the mobility trends in turkeys (Figs. 6 and 10), indicating a unique relationship between heterocoelous vertebrae and joint function. Nonetheless, the turkey neck form-function relationship is consistent with some trends found in other clades. Jones [70] measured greater axial rotation in horse vertebrae with more horizontal zygapophyses, which holds for the turkey data as well (Figs. 6, 8d and 10). Molnar et al. [18] found a positive correlation between crocodile prezygapophyseal width and lateroflexion; our regression results agree with this (Tables 1 and 2) and comparisons between prezygapophyseal width (Fig. 8a, b) and maximum lateroflexion excursion (Fig. 10) show similar patterns of variation.

## Conclusion

Although the primary goal of this study was to describe cervical joint function in the avian neck, the data have broader implications for understanding vertebral joint function more generally. The techniques employed here provide a repeatable protocol for any study examining the interactions between serially repeating segments with a large number of articulating joints. These methods enable detailed study of intact joints, and will hopefully serve as a foundation for future work on extant and fossil taxa that will be directly comparable. For example, the data presented here provide a framework for our larger study of the evolution of neck morphology and function along the line from non-avian dinosaurs to birds. Addressing our initial questions, our data demonstrate that: 1) The traditional three region model of avian necks may be present in turkeys, but these regions are somewhat ambiguous. There are cranial joints that are mobile in axial rotation and are relatively ventroflexed, caudal joints that have high lateroflexion ROM and little axial rotation, and middle joints that are transitional. However, there is variation within these regions that simple descriptions do not easily capture. 2) Substantial axial rotation can occur at joints caudal to the atlas/axis, contradicting common thought on heterocoelous joint function. 3) To achieve complex poses, zygapophyses can reduce their overlap almost to osteological disarticulation, providing evidence that ROM estimates using 25-50% overlap boundary conditions in cadaveric and fossil studies are likely conservative estimates. 4) Degrees of freedom interact at cervical joints; maximum lateroflexion occurs at different dorsoventral flexion angles at different joints, and axial rotation and lateroflexion are strongly coupled. These interactions should inform future analyses of cervical joint function and help constrain and guide reconstructions of neck poses in both extant and extinct animals. Finally, cervical morphology is significantly correlated to neck mobility, which should provide a framework for estimating neck function in skeletal material.

## Additional files

Additional file 1: Linear and angular measurements taken on CT scans of the 5 individuals used in this study. Measurements have been standardized by volume. (XLSX 27 kb)

Additional file 3: Appendix 1: Views of C3 and C9 comparing prezygapophyses. Appendix 2: Principal component scores for each of the joints sampled in the study based on an analysis of the ROM results. Appendix 3: Results of regressing range of motion on linear and angular measurements. (DOCX 199 kb)
