# Supplementary material for: Experimental determination of three-dimensional cervical joint mobility in the avian neck
Source: Front Zool. 2017 Jul 24;14:37. doi: 10.1186/s12983-017-0223-z (PMC5525307; doi:10.1186/s12983-017-0223-z)
Supplement: Supplementary file 3 — Appendix 1: Views of C3 and C9 comparing prezygapophyses. Appendix 2: Principal component scores for each of the joints sampled in the study based on an analysis of the ROM results. Appendix 3: Results of regressing range of motion on linear and angular measurements. (DOCX 199 kb) [file 12983_2017_223_MOESM3_ESM.docx]

Additional file 3: Experimental determination of three-dimensional cervical joint mobility in the avian neck

*Robert E. Kambic^1,2^, Andrew A. Biewener^2^, *Stephanie E. Pierce^1^

^1^Museum of Comparative Zoology and Department of Organismic and Evolutionary Biology, Harvard University, Cambridge, MA, 02138, USA.

^2^Concord Field Station, Department of Organismic and Evolutionary Biology, Harvard University, Bedford, MA 01730, USA.

robert.kambic@gmail.com

abiewener@oeb.harvard.edu

spierce@oeb.harvard.edu

*corresponding authors

Appendix 1: Craniolateral view of C3 and C9. CT scans of C3 and C9 rendered, showing the forward-pitched prezygapophyses of C3. Prezygapophyses boxed in red.


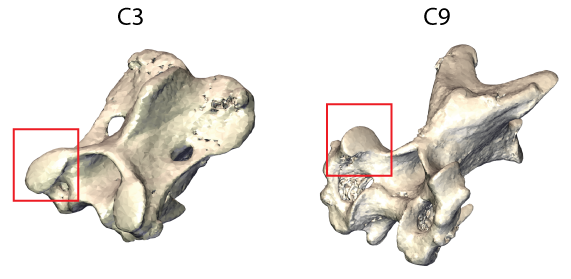


**Appendix 2**: Principal component analysis of maximum and minimum ROM for the six sampled joints. Analysis was run on maximum and minimum angle for dorsoventral flexion, lateroflexion, and axial rotation using the correlation matrix. Joints are plotted by score on the first two principal components, which accounted for 77% and 19% of total variation, respectively.


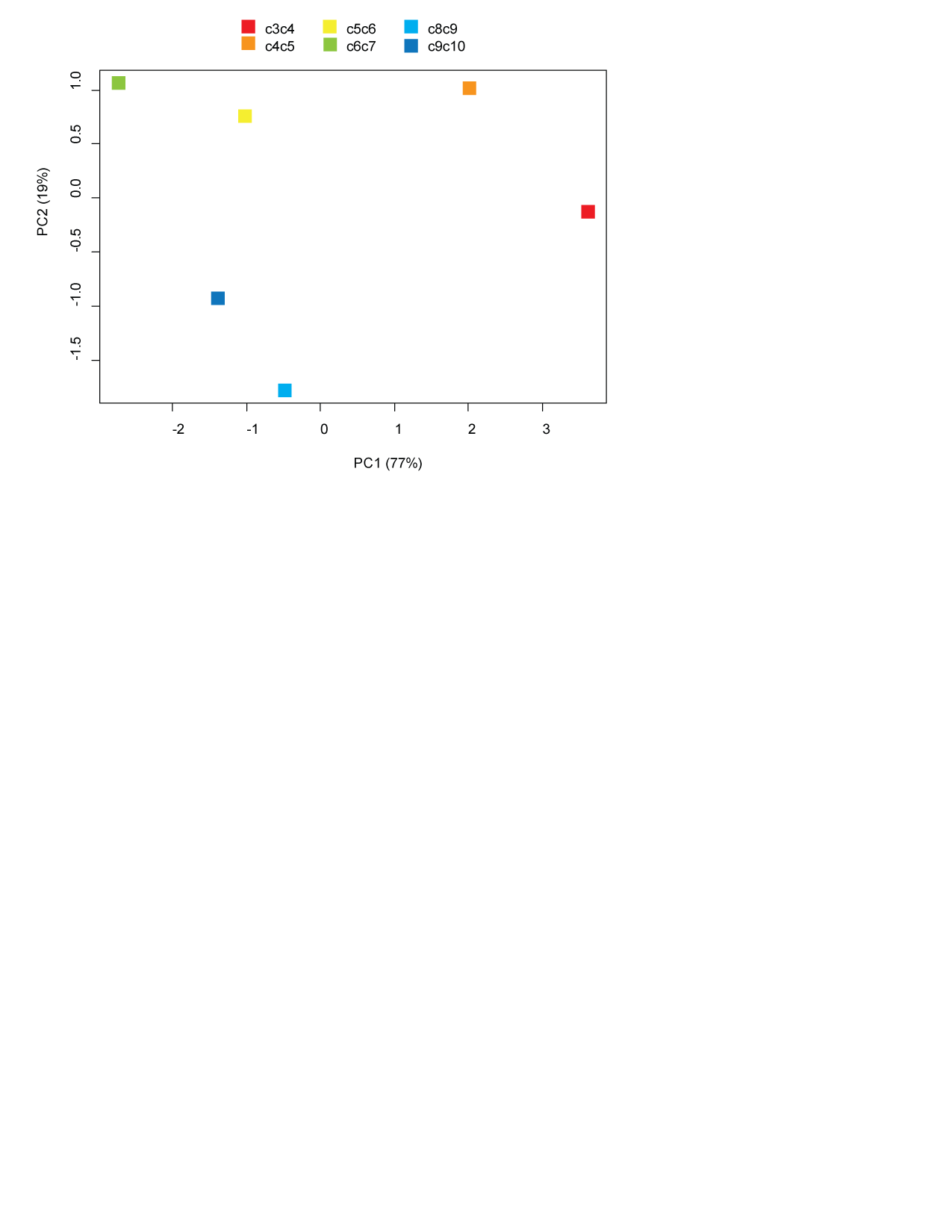


**Appendix 3**: Results of regressing range of motion on measurements. Volume-standardized linear measurements and raw angular measurements were regressed against axial rotation, lateroflexion, and mean dorsoventral excursion for the joint cranial and caudal to the vertebra.

Cranial Axial Rotation Excursion

Coefficients:

Estimate Std. Error t value Pr(>|t|)

(Intercept) 49.76422 14.32161 3.475 0.001961 **

Centrum.length -785.48902 136.33673 -5.761 6.14e-06 ***

Cranial.centrum.width -733.78584 258.88390 -2.834 0.009165 **

Cranial.zygapophyseal.width 744.55733 162.96589 4.569 0.000124 ***

Cranial.zygapophyseal.angle 0.09387 0.03052 3.076 0.005182 **

Caudal.zygapophyseal.angle 0.07581 0.04437 1.709 0.100426

---

Signif. codes: 0 ‘***’ 0.001 ‘**’ 0.01 ‘*’ 0.05 ‘.’ 0.1

Adjusted R-squared: 0.9316

Cranial Lateroflexion Excursion

Coefficients:

Estimate Std. Error t value Pr(>|t|)

(Intercept) 2.079e+01 2.593e+01 0.802 0.43182

Centrum.length -6.884e+02 2.061e+02 -3.340 0.00310 **

Vertebral.height 8.758e+02 4.201e+02 2.084 0.04952 *

Cranial.centrum.width -1.067e+03 4.975e+02 -2.145 0.04376 *

Cranial.zygapophyseal.angle 4.512e-02 3.603e-02 1.252 0.22423

Caudal.centrum.width 1.073e+03 8.649e+02 1.240 0.22851

Caudal.centrum.height -1.055e+03 6.279e+02 -1.679 0.10789

Caudal.zygapophyseal.width 1.098e+03 3.742e+02 2.934 0.00794 **

Caudal.zygapophyseal.angle 1.441e-01 7.953e-02 1.812 0.08439 .

---

Signif. codes: 0 ‘***’ 0.001 ‘**’ 0.01 ‘*’ 0.05 ‘.’ 0.1

Adjusted R-squared: 0.6859

Cranial Mean Dorsoventral flexion

Coefficients:

Estimate Std. Error t value Pr(>|t|)

(Intercept) -1.895e+01 1.428e+01 -1.327 0.19747

Centrum.length 5.149e+02 1.489e+02 3.459 0.00213 **

Cranial.centrum.width 1.472e+03 3.990e+02 3.690 0.00121 **

Cranial.centrum.height 1.220e+03 5.125e+02 2.381 0.02595 *

Cranial.zygapophyseal.angle -1.582e-01 2.854e-02 -5.543 1.23e-05 ***

Caudal.centrum.width -1.823e+03 5.787e+02 -3.150 0.00448 **

Caudal.centrum.height -7.707e+02 4.148e+02 -1.858 0.07605 .

---

Signif. codes: 0 ‘***’ 0.001 ‘**’ 0.01 ‘*’ 0.05 ‘.’ 0.1

Adjusted R-squared: 0.9259

Caudal Axial Rotation Excursion

Coefficients:

Estimate Std. Error t value Pr(>|t|)

(Intercept) 61.48 14.14 4.348 0.000258 ***

Centrum.length -625.39 180.57 -3.463 0.002210 **

Cranial.centrum.width 927.24 525.55 1.764 0.091559 .

Cranial.centrum.height -989.61 687.31 -1.440 0.163998

Cranial.zygapophyseal.width -561.55 228.49 -2.458 0.022334 *

Caudal.centrum.width -746.73 600.54 -1.243 0.226796

Caudal.centrum.height -1045.87 515.10 -2.030 0.054573 .

Caudal.zygapophyseal.width 1578.59 246.50 6.404 1.92e-06 ***

---

Signif. codes: 0 ‘***’ 0.001 ‘**’ 0.01 ‘*’ 0.05 ‘.’ 0.1

Adjusted R-squared: 0.9051

Caudal Lateroflexion Excursion

Coefficients:

Estimate Std. Error t value Pr(>|t|)

(Intercept) 47.08 11.29 4.169 0.000321 ***

Cranial.centrum.width 1699.34 746.76 2.276 0.031701 *

Caudal.centrum.width -2248.58 775.49 -2.900 0.007676 **

Caudal.centrum.height -1266.19 706.60 -1.792 0.085252 .

Caudal.zygapophyseal.width 1051.07 253.93 4.139 0.000346 ***

---

Signif. codes: 0 ‘***’ 0.001 ‘**’ 0.01 ‘*’ 0.05 ‘.’ 0.1

Adjusted R-squared: 0.4747

Caudal Mean Dorsoventral flexion

Coefficients:

Estimate Std. Error t value Pr(>|t|)

(Intercept) -30.22 12.78 -2.365 0.026447 *

Centrum.length 618.86 153.75 4.025 0.000495 ***

Cranial.centrum.height 1016.02 607.35 1.673 0.107339

Cranial.zygapophyseal.width -558.57 200.45 -2.787 0.010242 *

Caudal.centrum.height 637.75 438.64 1.454 0.158918

Caudal.zygapophyseal.width -385.65 220.74 -1.747 0.093410 .

---

Signif. codes: 0 ‘***’ 0.001 ‘**’ 0.01 ‘*’ 0.05 ‘.’ 0.1

Adjusted R-squared: 0.9266
